# Supplementary material for: SoxB1-Mediated Chromatin Remodeling Promotes Sensory Neuron Differentiation in Planarians
Source: bioRxiv. 2025 Dec 2:2025.09.01.673518. Originally published 2025 Sep 2. Preprint. [Version 2] doi: 10.1101/2025.09.01.673518 (PMC12424676; doi:10.1101/2025.09.01.673518)

bioRxiv preprint doi: https://doi.org/10.1101/2025.09.01.673518; this version posted December 2, 2025. The copyright holder for this preprint (which was not certified by peer review) is the author/funder, who has granted bioRxiv a license to display the preprint in perpetuity. It is made available under aCC-BY 4.0 International license.

SUPPLEMENTAL FIGURE 1

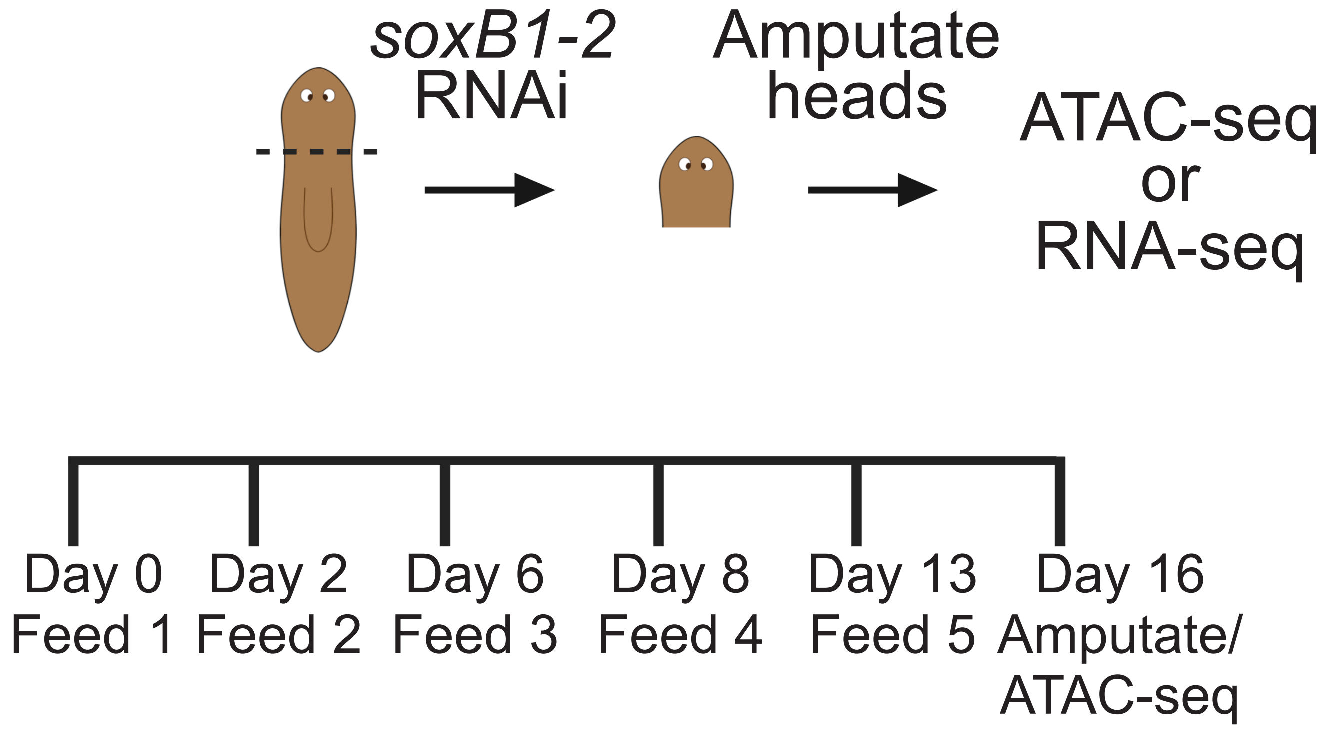

bioRxiv preprint doi: https://doi.org/10.1101/2025.09.01.673518; this version posted December 2, 2025. The copyright holder for this preprint (which was not certified by peer review) is the author/funder, who has granted bioRxiv a license to display the preprint in perpetuity. It is made available under aCC-BY 4.0 International license.

SUPPLEMENTAL FIGURE 2

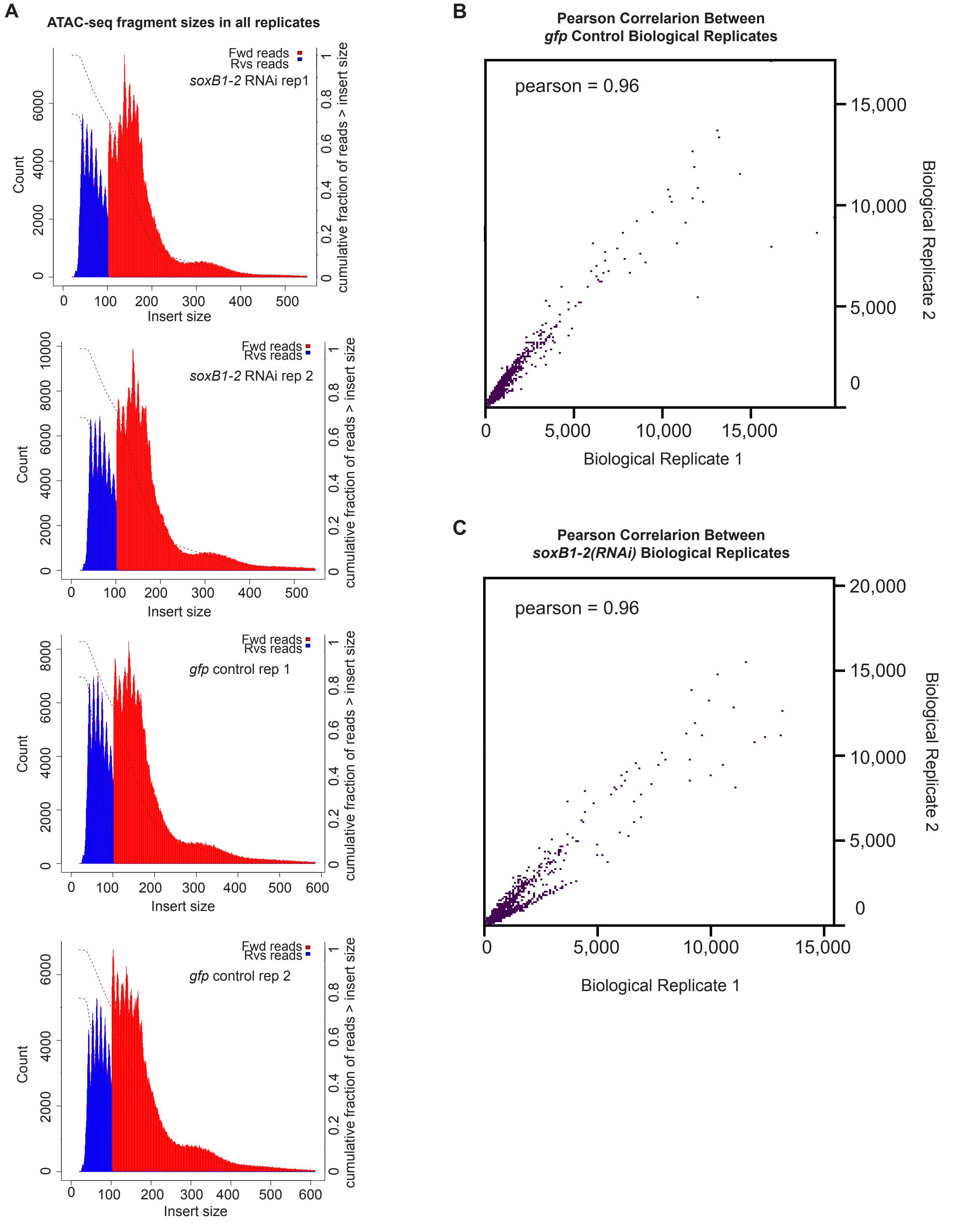

bioRxiv preprint doi: https://doi.org/10.1101/2025.09.01.673518; this version posted December 2, 2025. The copyright holder for this preprint (which was not certified by peer review) is the author/funder, who has granted bioRxiv a license to display the preprint in perpetuity. It is made available under aCC-BY 4.0 International license.

SUPPLEMENTAL FIGURE 3

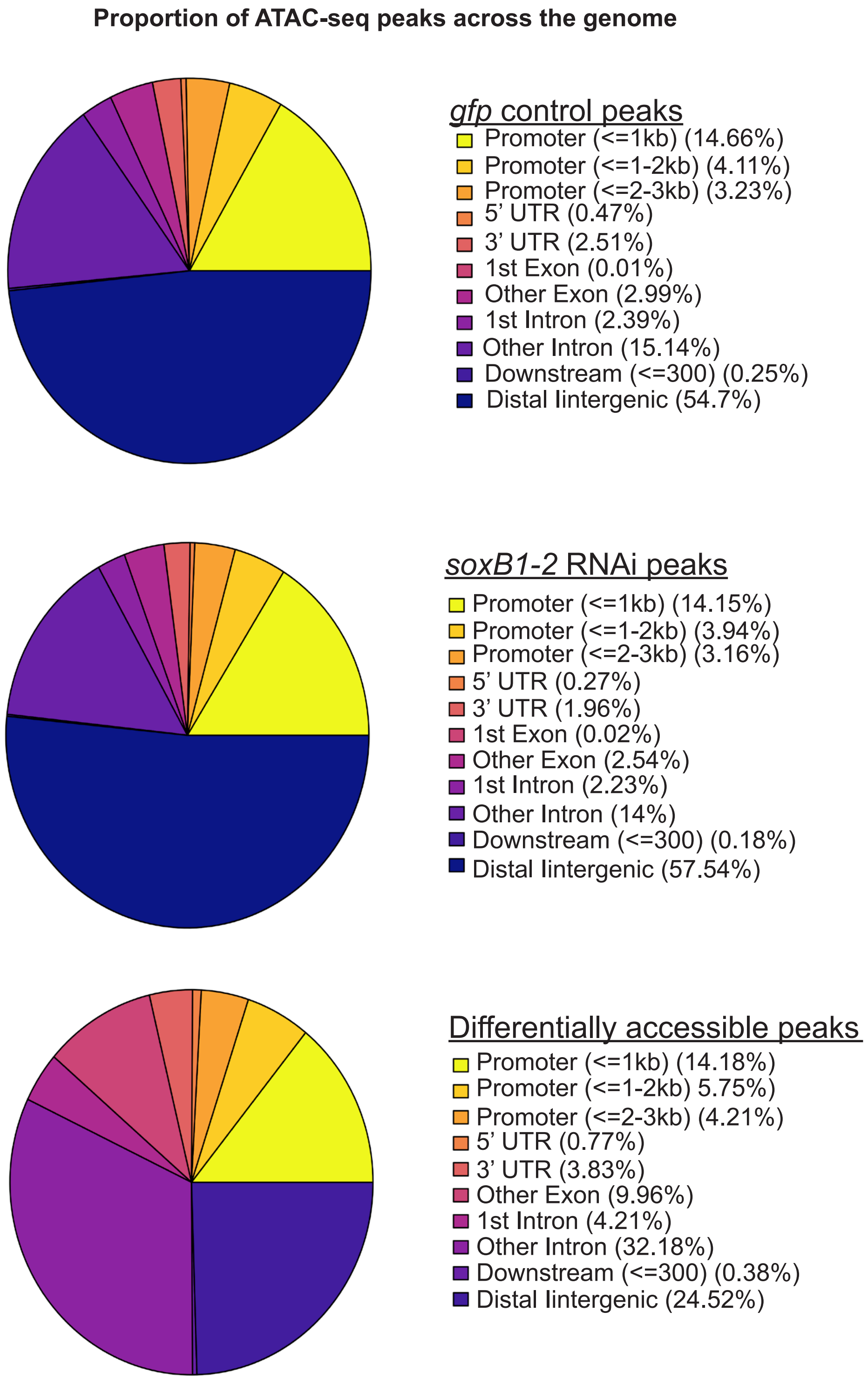

bioRxiv preprint doi: https://doi.org/10.1101/2025.09.01.673518; this version posted December 2, 2025. The copyright holder for this preprint (which was not certified by peer review) is the author/funder, who has granted bioRxiv a license to display the preprint in perpetuity. It is made available under aCC-BY 4.0 International license.

SUPPLEMENTAL FIGURE 4

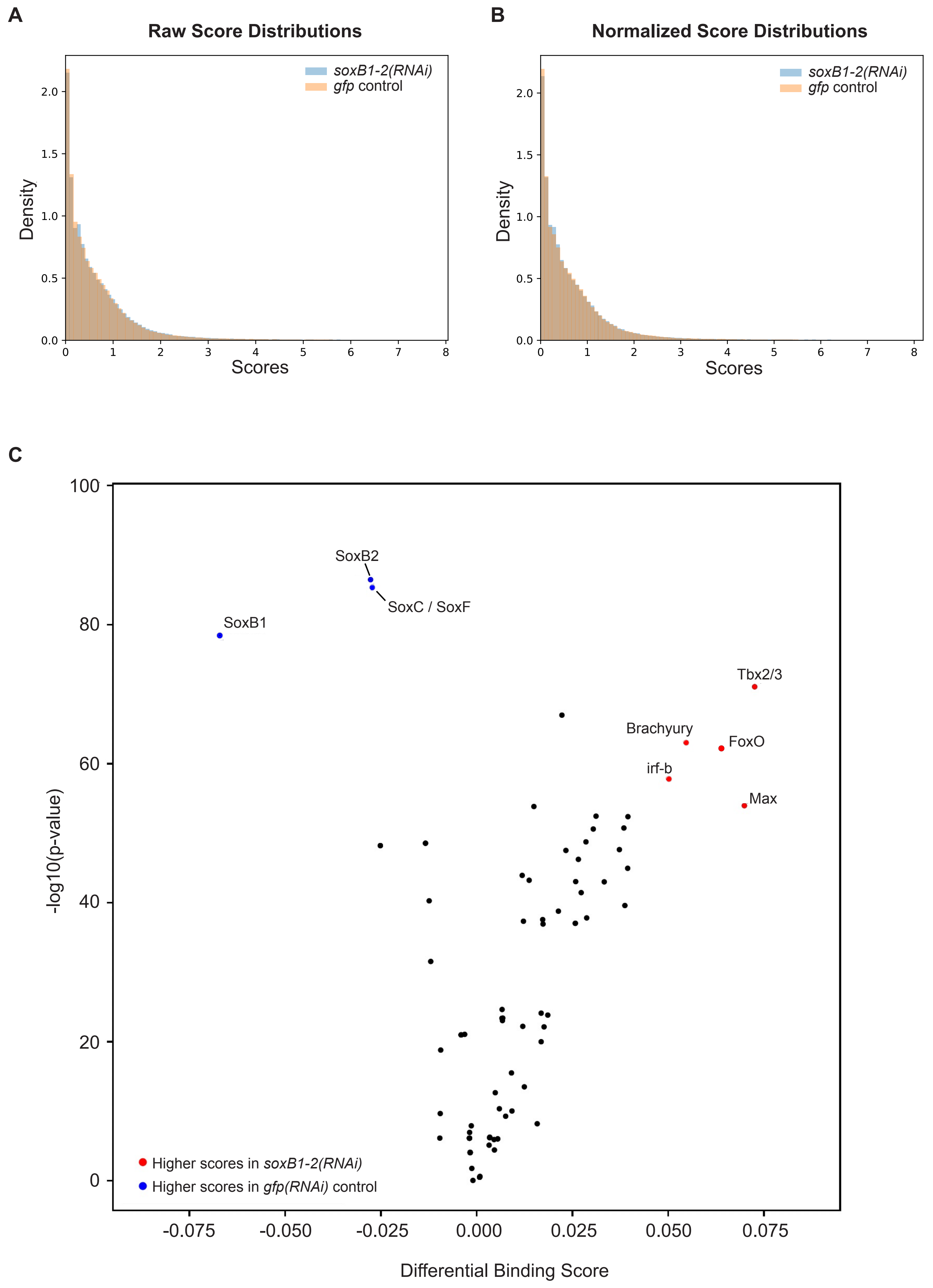

bioRxiv preprint doi: https://doi.org/10.1101/2025.09.01.673518; this version posted December 2, 2025. The copyright holder for this preprint (which was not certified by peer review) is the author/funder, who has granted bioRxiv a license to display the preprint in perpetuity. It is made available under aCC-BY 4.0 International license.

SUPPLEMENTAL FIGURE 5

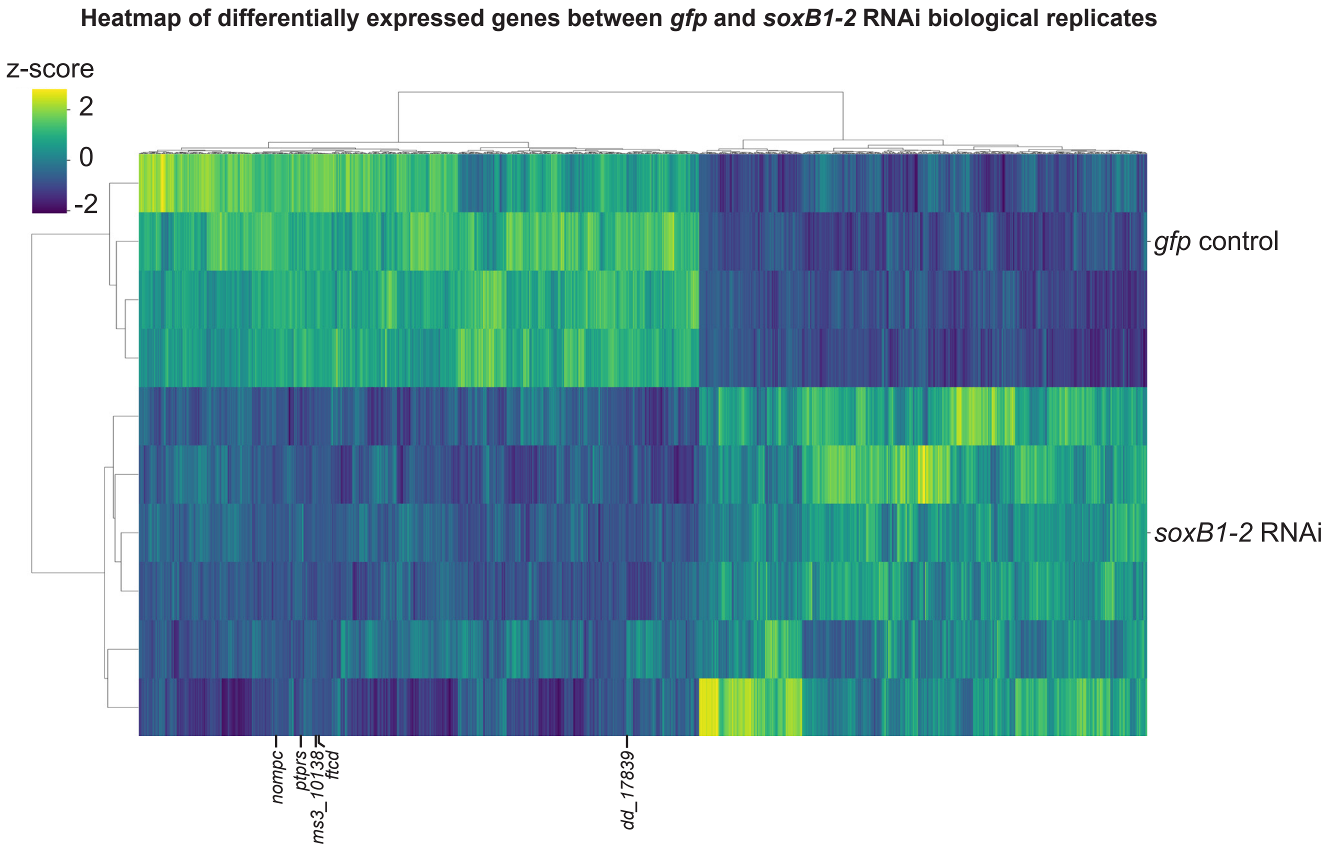

bioRxiv preprint doi: https://doi.org/10.1101/2025.09.01.673518; this version posted December 2, 2025. The copyright holder for this preprint (which was not certified by peer review) is the author/funder, who has granted bioRxiv a license to display the preprint in perpetuity. It is made available under aCC-BY 4.0 International license.

SUPPLEMENTAL FIGURE 6

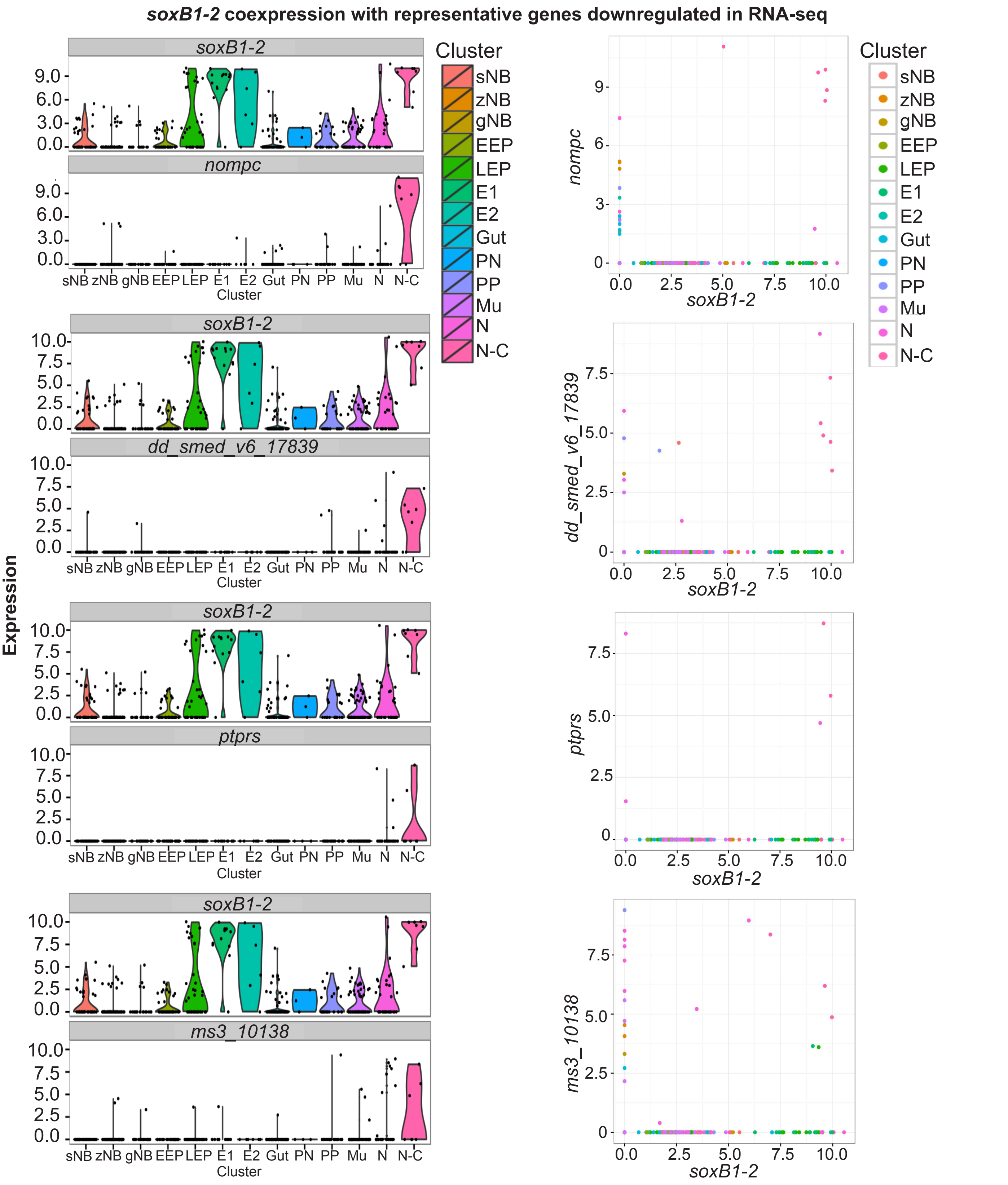

bioRxiv preprint doi: https://doi.org/10.1101/2025.09.01.673518; this version posted December 2, 2025. The copyright holder for this preprint (which was not certified by peer review) is the author/funder, who has granted bioRxiv a license to display the preprint in perpetuity. It is made available under aCC-BY 4.0 International license.

SUPPLEMENTAL FIGURE 7

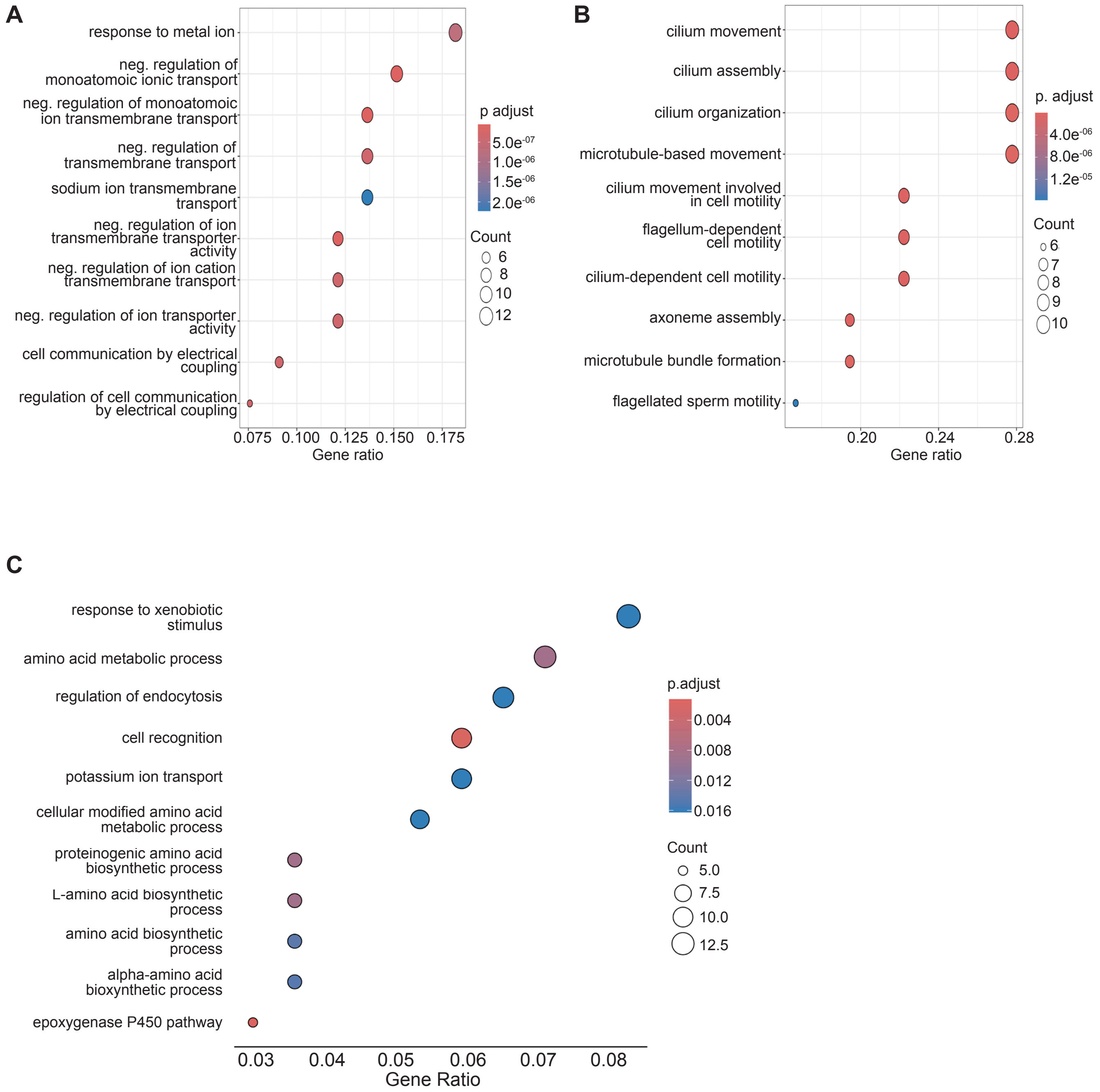

bioRxiv preprint doi: https://doi.org/10.1101/2025.09.01.673518; this version posted December 2, 2025. The copyright holder for this preprint (which was not certified by peer review) is the author/funder, who has granted bioRxiv a license to display the preprint in perpetuity. It is made available under aCC-BY 4.0 International license.

SUPPLEMENTAL FIGURE 8

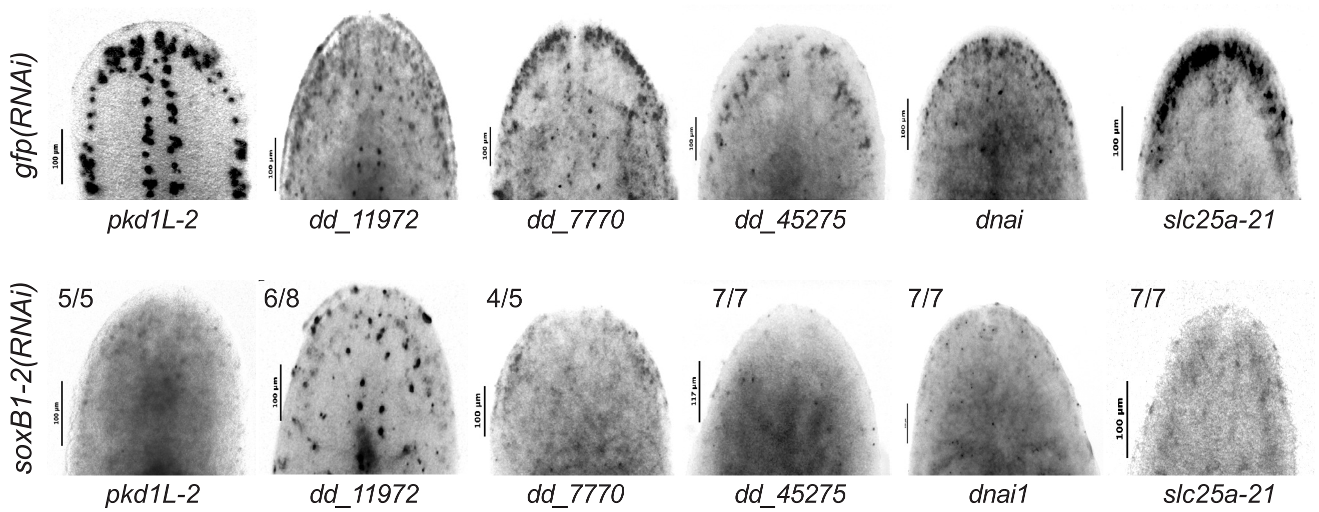

bioRxiv preprint doi: https://doi.org/10.1101/2025.09.01.673518; this version posted December 2, 2025. The copyright holder for this preprint (which was not certified by peer review) is the author/funder, who has granted bioRxiv a license to display the preprint in perpetuity. It is made available under aCC-BY 4.0 International license.

SUPPLEMENTAL FIGURE 9

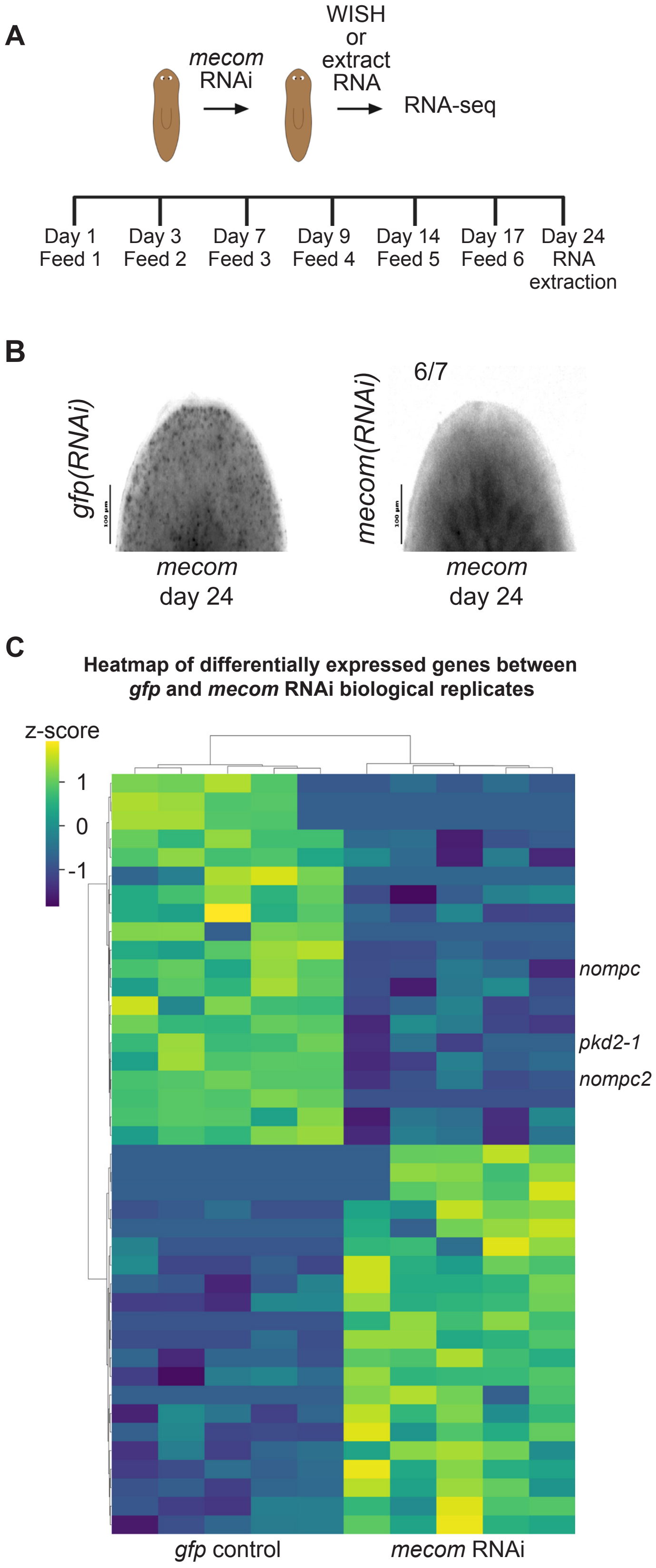

bioRxiv preprint doi: https://doi.org/10.1101/2025.09.01.673518; this version posted December 2, 2025. The copyright holder for this preprint (which was not certified by peer review) is the author/funder, who has granted bioRxiv a license to display the preprint in perpetuity. It is made available under aCC-BY 4.0 International license.

SUPPLEMENTAL FIGURE 10

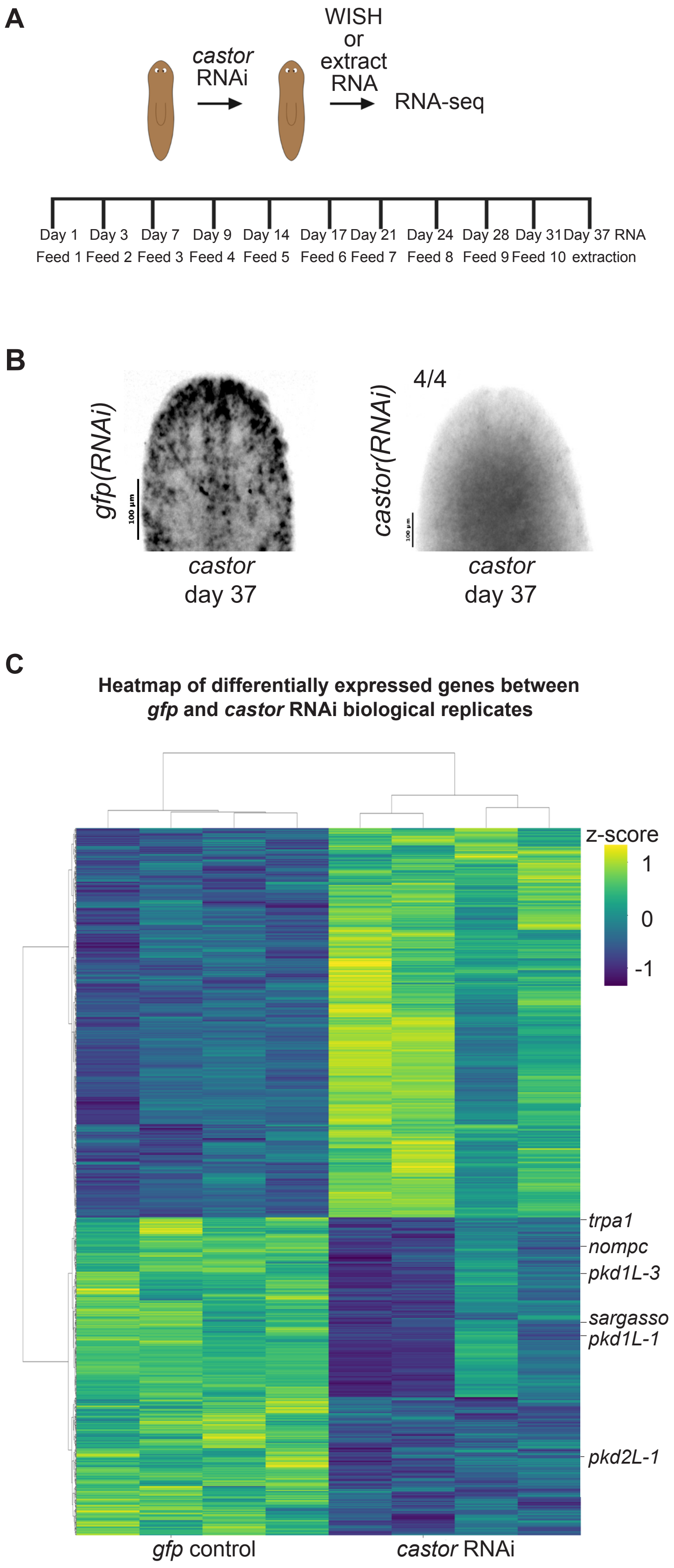

Supplement: Supplement 6 — Supplemental Figure 1. Schematic of the assay used to assess chromatin accessibility and transcriptional changes in gfp control and soxB1-2 RNAi conditions. Supplemental Figure 2. Fragment size distribution and Pearson correlation analysis of ATAC-seq replicates. (A) Fragment size distributions from each ATAC-seq replicate. Forward and reverse reads are shown in red and blue, respectively. (B) Biological replicates within control groups are highly correlated (r = 0.96; n = 2). (C) Biological replicates within soxB1-2 RNAi groups are highly correlated (r = 0.96; n = 2). Supplemental Figure 3. ATAC-seq analysis reveals differences in chromatin accessibility following soxB1-2 knockdown. Distribution of ATAC-seq peaks across annotated genomic regions of the S. mediterranea genome, comparing all gfp control and soxB1-2 RNAi replicates. "DA peaks" indicate regions with detectable accessibility in gfp controls that are absent following soxB1-2 knockdown. Supplemental Figure 4. Motif enrichment analysis of differential ATAC-seq footprints using BINDetect. (A) Raw score distributions for transcription factor binding site footprints in control and soxB1-2 RNAi samples, showing the density of unscaled footprint scores across conditions. (B) Normalized score distributions after within-condition scaling, demonstrating comparable score distributions suitable for differential binding analysis. (C) Volcano plot of differential transcription factor binding generated by BINDetect. Positive differential binding scores indicate motifs with higher footprint occupancy in soxB1-2 RNAi samples, whereas negative scores indicate motifs more enriched in control samples. Notably, Sox family motifs (SoxB1, SoxB2, SoxC, SoxF) and several T-box factors (e.g., Tbx2/3, Brachyury) show strong differential binding. Supplemental Figure 5. Heatmap of isolated differentially expressed genes (FC ≥ 1.4, p < 0.05) in gfp control vs. soxB1-2 RNAi groups. Z-scores indicate upregulation (2), no change (0), an [file NIHPP2025.09.01.673518v2-supplement-6.pdf]
