# Supplementary material for: SoxB1-Mediated Chromatin Remodeling Promotes Sensory Neuron Differentiation in Planarians
Source: bioRxiv. 2025 Sep 2:2025.09.01.673518. Preprint. [Version 1] doi: 10.1101/2025.09.01.673518 (PMC12424676; doi:10.1101/2025.09.01.673518)

bioRxiv preprint doi: https://doi.org/10.1101/2025.09.01.673518; this version posted September 2, 2025. The copyright holder for this preprint (which was not certified by peer review) is the author/funder, who has granted bioRxiv a license to display the preprint in perpetuity. It is made available under aCC-BY 4.0 International license.

Supplemental Figure Legends

Supplemental Figure S1. ATAC-seq analysis reveals differences in chromatin accessibility following *soxB1-2* knockdown. (A) Schematic of the assay used to assess chromatin accessibility in *gfp* control and *soxB1-2* RNAi conditions. (B) Distribution of ATAC-seq peaks across annotated genomic regions of the *S. mediterranea* genome, comparing all *gfp* control and *soxB1-2* RNAi replicates. "DA peaks" indicate regions with detectable accessibility in *gfp* controls that are absent following *soxB1-2* knockdown. (C) Fragment size distributions from each ATAC-seq replicate. Forward and reverse reads are shown in red and blue, respectively.

Supplemental Figure S2. Co-expression of *soxB1-2* and select genes identified by RNA-seq. (A) Single-cell expression profiles of cells co-expressing *soxB1-2* and representative RNA-seq–identified genes that are significantly downregulated following *soxB1-2* RNAi. Cell type abbreviations: sNB, sigma neoblast; zNB, zeta neoblast; gNB, gamma neoblast; EEP, early epidermal progenitor; LEP, late epidermal progenitor; E1, epidermis 1; E2, epidermis 2; Gut, intestine; PN, protonephridia; PP, parapharyngeal; Mu, muscle; N, neural; N-C, neural-ciliated. (B) Gene Ontology (GO) term enrichment of 134 genes shared between RNA-seq datasets from whole-worm and head-only samples. (C) Gene Ontology (GO) term enrichment of 148 downregulated genes uniquely identified in whole-worm RNA-seq datasets. (D) Heatmap of isolated differentially expressed genes ($FC \geq 1.4$, $p < 0.05$) in *gfp* control vs. *soxB1-2* RNAi groups. Z-scores indicate upregulation (2), no change (0), and downregulation (-2). Labeled genes represent putative sensory neural genes selected for validation via in situ hybridization.

bioRxiv preprint doi: https://doi.org/10.1101/2025.09.01.673518; this version posted September 2, 2025. The copyright holder for this preprint (which was not certified by peer review) is the author/funder, who has granted bioRxiv a license to display the preprint in perpetuity. It is made available under aCC-BY 4.0 International license.

Supplemental Figure S3. Expression patterns of genes with reduced chromatin accessibility or transcription following *soxB1-2* RNAi, and confirmation of *mecom* transcript loss after RNAi. (A) Decreased or absent expression of genes identified through ATAC-seq or RNA-seq as downregulated following *soxB1-2* RNAi. *dd_11972* shows reduced expression in the head tip and across the epidermis, while *dd_7770*, *dd_45275*, *dnai1*, and s*lc25a-21* exhibit complete loss of expression. *pkd1L-2* serves as a positive control for confirming the knockdown of *soxB1-2* downstream targets via in situ hybridization. (B) Expression of genes enriched in the central nervous system that appeared in ATAC-seq or RNA-seq datasets but did not meet statistical cut-offs, yet exhibited reduced brain size phenotypes. *dd_2708* additionally showed loss of expression in sensory cells. Black arrows indicate regions with reduced or absent expression. (C) Validation of *mecom* transcript loss at the time point used for RNA-seq sample collection. (D) Validation of *castor* transcript loss at the time point used for RNA-seq sample collection. Sample size: For controls, $N \geq 3$ worms were analyzed, and all samples showed consistent expression patterns. For RNAi-treated animals, the number of biological replicates (N) is indicated at the top left of each treatment group.

Supplemental Figure S4. Experimental design for *mecom* RNA-seq and heatmap of candidate targets meeting differential expression cut-offs. (A) Schematic of the assay used to assess differential gene expression in *gfp* control and *mecom* RNAi conditions. (B) Heatmap of differentially expressed genes ($FC \geq 1.4$, $p < 0.1$) in *gfp* control vs. *mecom* RNAi groups. Z-scores indicate upregulation (2), no change (0), and downregulation (-2). Labeled genes are

bioRxiv preprint doi: https://doi.org/10.1101/2025.09.01.673518; this version posted September 2, 2025. The copyright holder for this preprint (which was not certified by peer review) is the author/funder, who has granted bioRxiv a license to display the preprint in perpetuity. It is made available under aCC-BY 4.0 International license.

putative mechanosensory genes identified in RNA-seq experiments performed on *mecom* RNAi samples.

Supplemental Figure S5. Experimental design for *castor* RNA-seq and heatmap of candidate targets meeting differential expression cut-offs. (A) Schematic of the assay used to assess differential gene expression in *gfp* control and *castor* RNAi conditions. (B) Heatmap of differentially expressed genes (FC $\geq$ 1.4, $p < 0.1$) in *gfp* control vs. *castor* RNAi groups. Z-scores indicate upregulation (2), no change (0), and downregulation (-2). Labeled genes are putative mechanosensory genes identified in RNA-seq experiments performed on *castor* RNAi samples.

bioRxiv preprint doi: https://doi.org/10.1101/2025.09.01.673518; this version posted September 2, 2025. The copyright holder for this preprint (which was not certified by peer review) is the author/funder, who has granted bioRxiv a license to display the preprint in perpetuity. It is made available under aCC-BY 4.0 International license.

SUPPLEMENTAL FIGURE 1

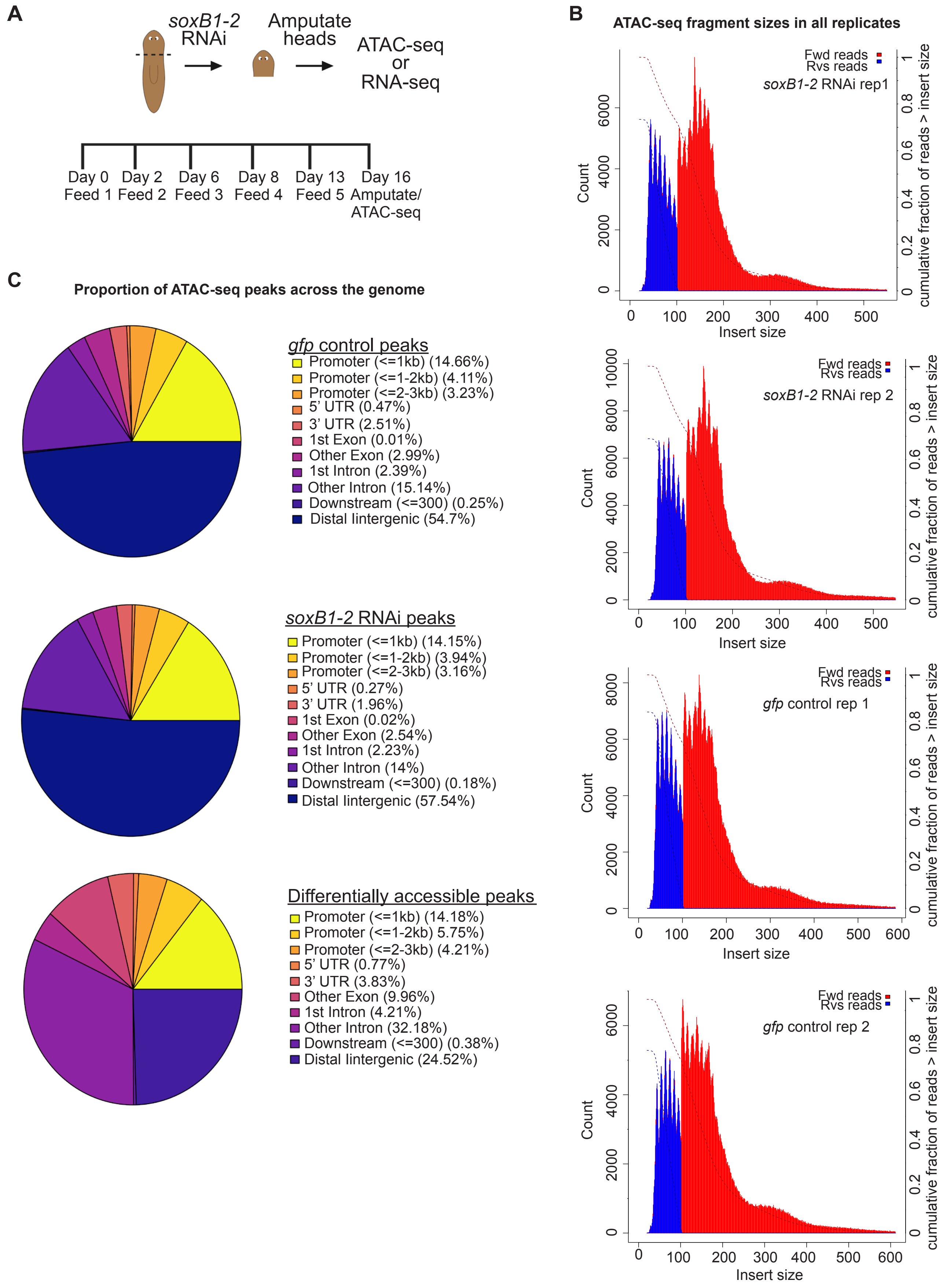

bioRxiv preprint doi: https://doi.org/10.1101/2025.09.01.673518; this version posted September 2, 2025. The copyright holder for this preprint (which was not certified by peer review) is the author/funder, who has granted bioRxiv a license to display the preprint in perpetuity. It is made available under aCC-BY 4.0 International license.

SUPPLEMENTAL FIGURE 2

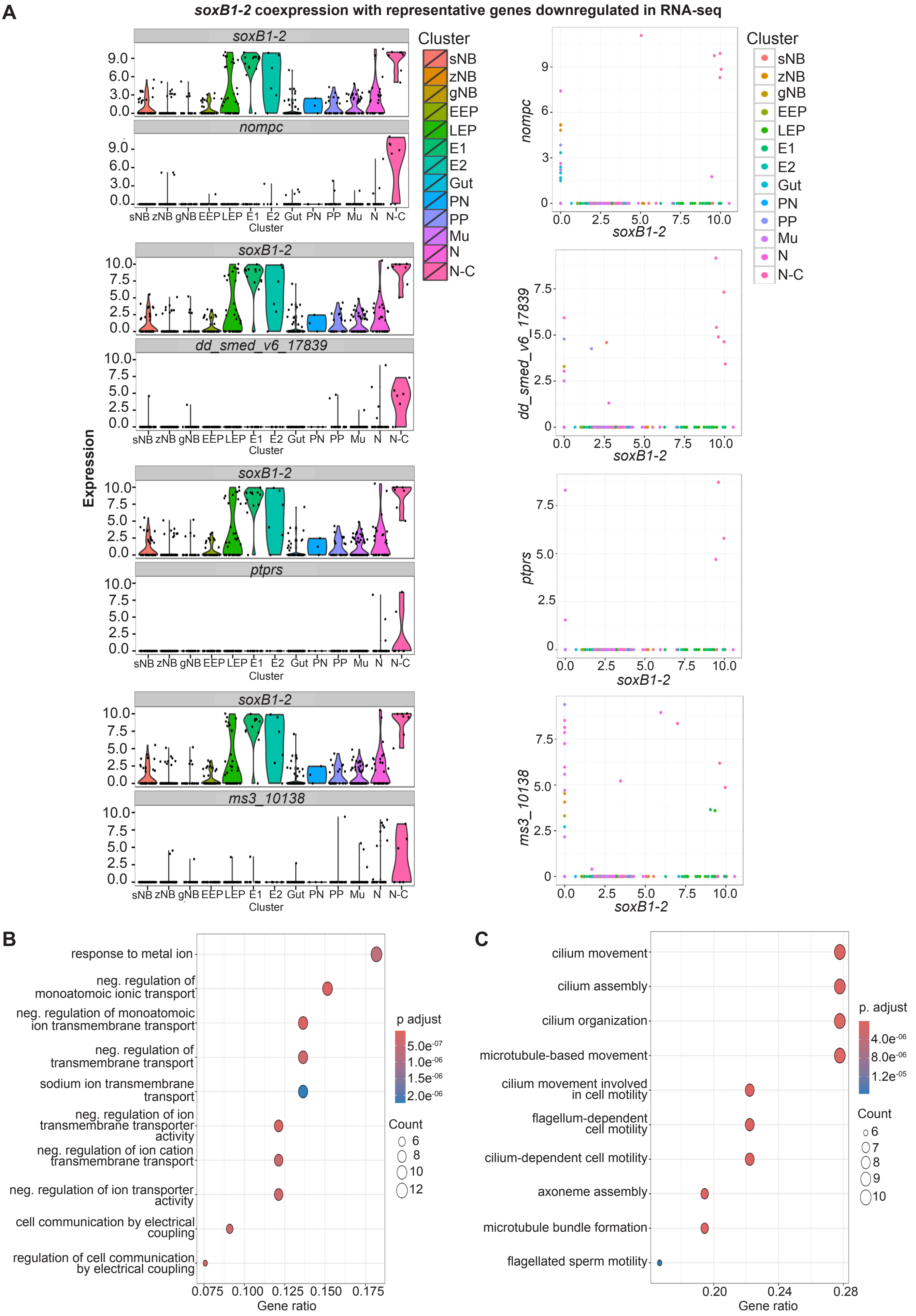

bioRxiv preprint doi: https://doi.org/10.1101/2025.09.01.673518; this version posted September 2, 2025. The copyright holder for this preprint (which was not certified by peer review) is the author/funder, who has granted bioRxiv a license to display the preprint in perpetuity. It is made available under aCC-BY 4.0 International license.

SUPPLEMENTAL FIGURE 2 (continued)

D

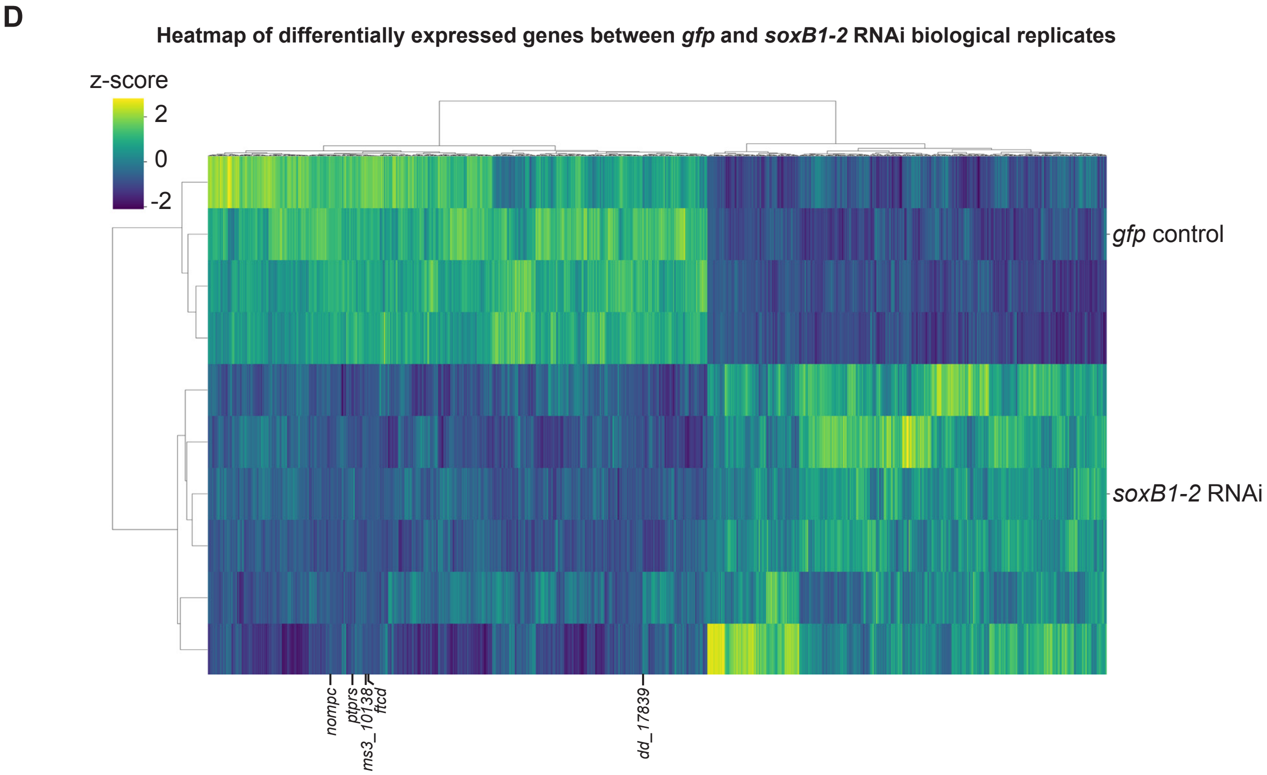

bioRxiv preprint doi: https://doi.org/10.1101/2025.09.01.673518; this version posted September 2, 2025. The copyright holder for this preprint (which was not certified by peer review) is the author/funder, who has granted bioRxiv a license to display the preprint in perpetuity. It is made available under aCC-BY 4.0 International license.

SUPPLEMENTAL FIGURE 3

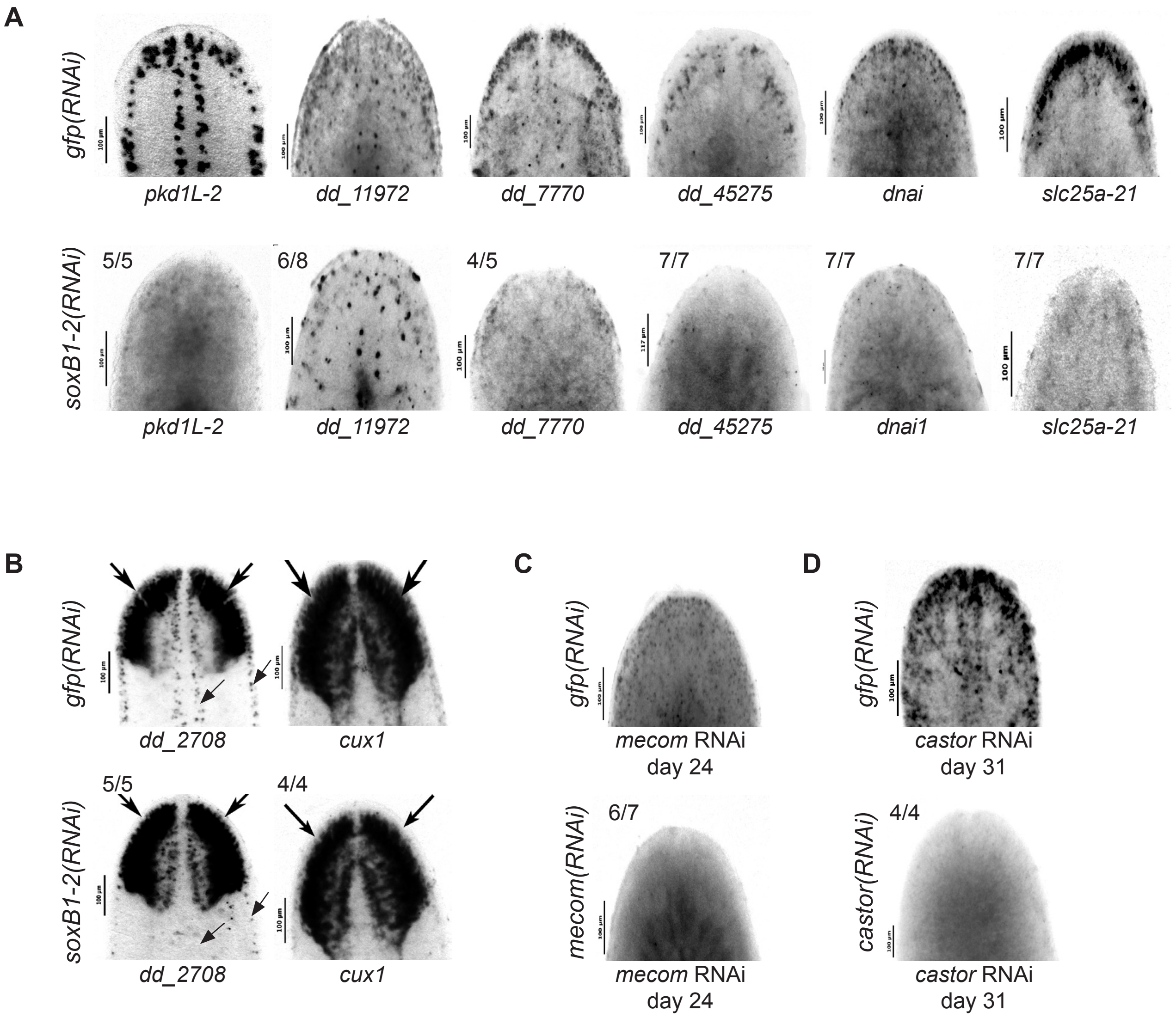

bioRxiv preprint doi: https://doi.org/10.1101/2025.09.01.673518; this version posted September 2, 2025. The copyright holder for this preprint (which was not certified by peer review) is the author/funder, who has granted bioRxiv a license to display the preprint in perpetuity. It is made available under aCC-BY 4.0 International license.

SUPPLEMENTAL FIGURE 4

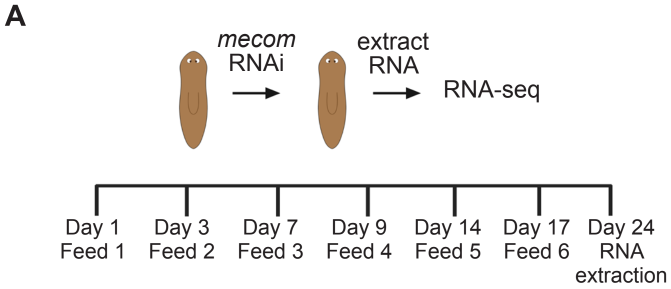

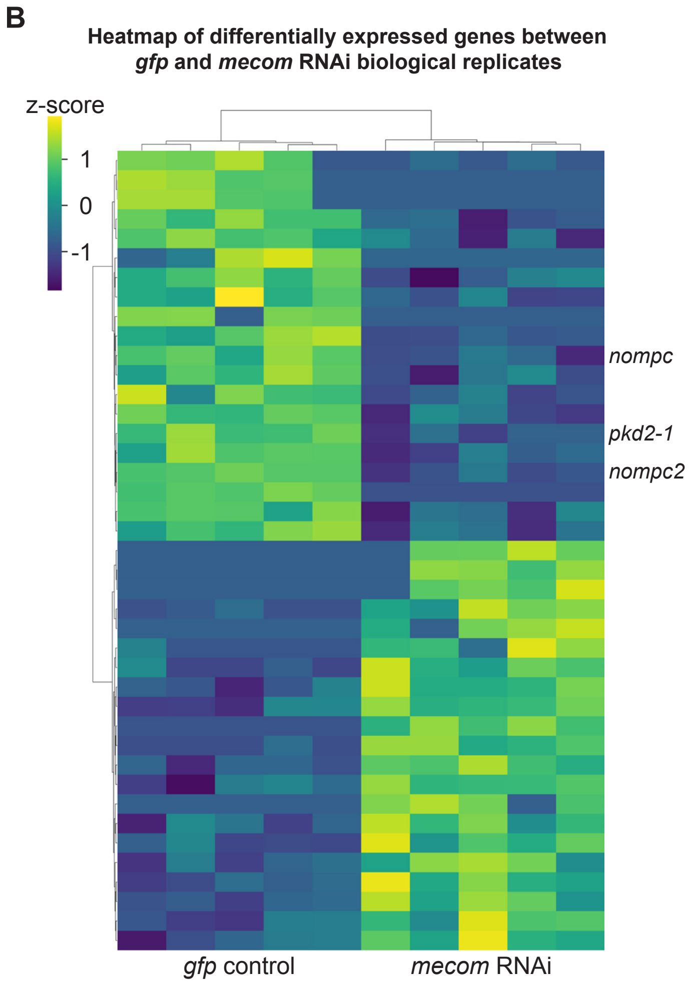

bioRxiv preprint doi: https://doi.org/10.1101/2025.09.01.673518; this version posted September 2, 2025. The copyright holder for this preprint (which was not certified by peer review) is the author/funder, who has granted bioRxiv a license to display the preprint in perpetuity. It is made available under aCC-BY 4.0 International license.

SUPPLEMENTAL FIGURE 5

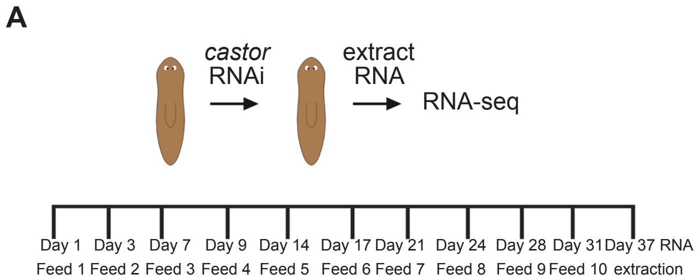

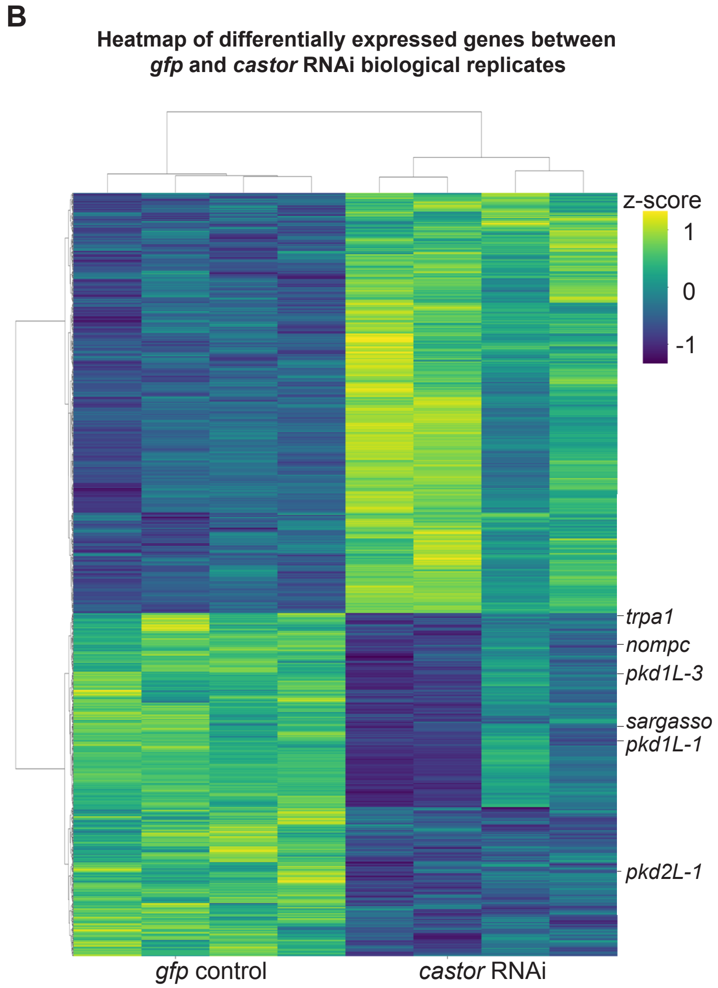

Supplement: Supplementary file 1 [file NIHPP2025.09.01.673518v1-supplement-6.pdf]
